# Psychometric Validation of the Japanese Version of the Neuropathic Pain Symptom Inventory

**DOI:** 10.1371/journal.pone.0143350

**Published:** 2015-11-24

**Authors:** Yoshitaka Matsubayashi, Katsushi Takeshita, Masahiko Sumitani, Yasushi Oshima, Juichi Tonosu, So Kato, Junichi Ohya, Takeshi Oichi, Naoki Okamoto, Sakae Tanaka

**Affiliations:** 1 Department of Orthopaedic Surgery, Faculty of Medicine, The University of Tokyo, Tokyo, Japan; 2 Department of Orthopaedic Surgery, Jichi Medical University, Tochigi, Japan; 3 Department of Pain and Palliative Medicine, The University of Tokyo, Tokyo, Japan; Tokyo Metropolitan Institute of Medical Science, JAPAN

## Abstract

**Objective:**

This study aimed to evaluate the validity and reliability of the Japanese version of the Neuropathic Pain Symptom Inventory (NPSI-J).

**Design:**

Cross-sectional study design.

**Subjects and Methods:**

The original Neuropathic Pain Symptom Inventory (NPSI) was translated into Japanese according to published guidelines. Subsequently, an observational study of 60 Japanese patients suffering from neuropathic pain was performed to evaluate the validity and reliability of the NPSI-J.

**Results:**

The NPSI-J exhibited a statistically significant correlation with pain intensity (Numerical Rating Scale). The Cronbach alpha value for Likert items was 0.86. Using the test–retest analysis method, the intraclass correlation coefficient between the two scores was 0.81. Factor analysis revealed that the main component of NPSI-J comprised three determinative factors.

**Conclusions:**

The NPSI-J is a reliable and valid pain assessment tool.

## Introduction

Neuropathic pain is more difficult to treat than many other types of chronic pain and therefore profoundly impairs the quality of life. In particular, patients with neuropathic pain are more likely to bear a greater burden of co-morbid disease relative to age- and gender-adjusted control subjects [1]. The International Association for the Study of Pain, Neuropathic Pain Special Interest Group has proposed a grading system for neuropathic pain, which is currently defined as “pain caused by a lesion or disease of the somatosensory nervous system” [2, 3]. However, this grading system can fail to diagnose some patients with neuropathic pain. Accordingly, research has been implemented to prevent false-negative diagnoses of neuropathic pain and support physicians’ decisions to initiate specific pharmacotherapeutic approaches through the development of screening tools, nearly all of which are based on verbal pain descriptions with or without limited bedside testing [4, 5].

The unique painful and non-painful sensations experienced by patients with neuropathic pain are widely believed to result from particular mechanisms, and ideally specific management strategies would be applied for such pain. During the course of treatment for neuropathic pain, precise evaluations of pain in terms of both quantity (intensity) and quality (pain characteristics) are essential. Neuropathic pain usually presents with characteristic symptoms, such as a “burning sensation,” “prickling sensation,” and/or a sensation of “electric shock,” and these characteristics support the subgrouping of etiologically diverse neuropathic pain patients into a specific multidimensional category [6].

**Fig 1:**
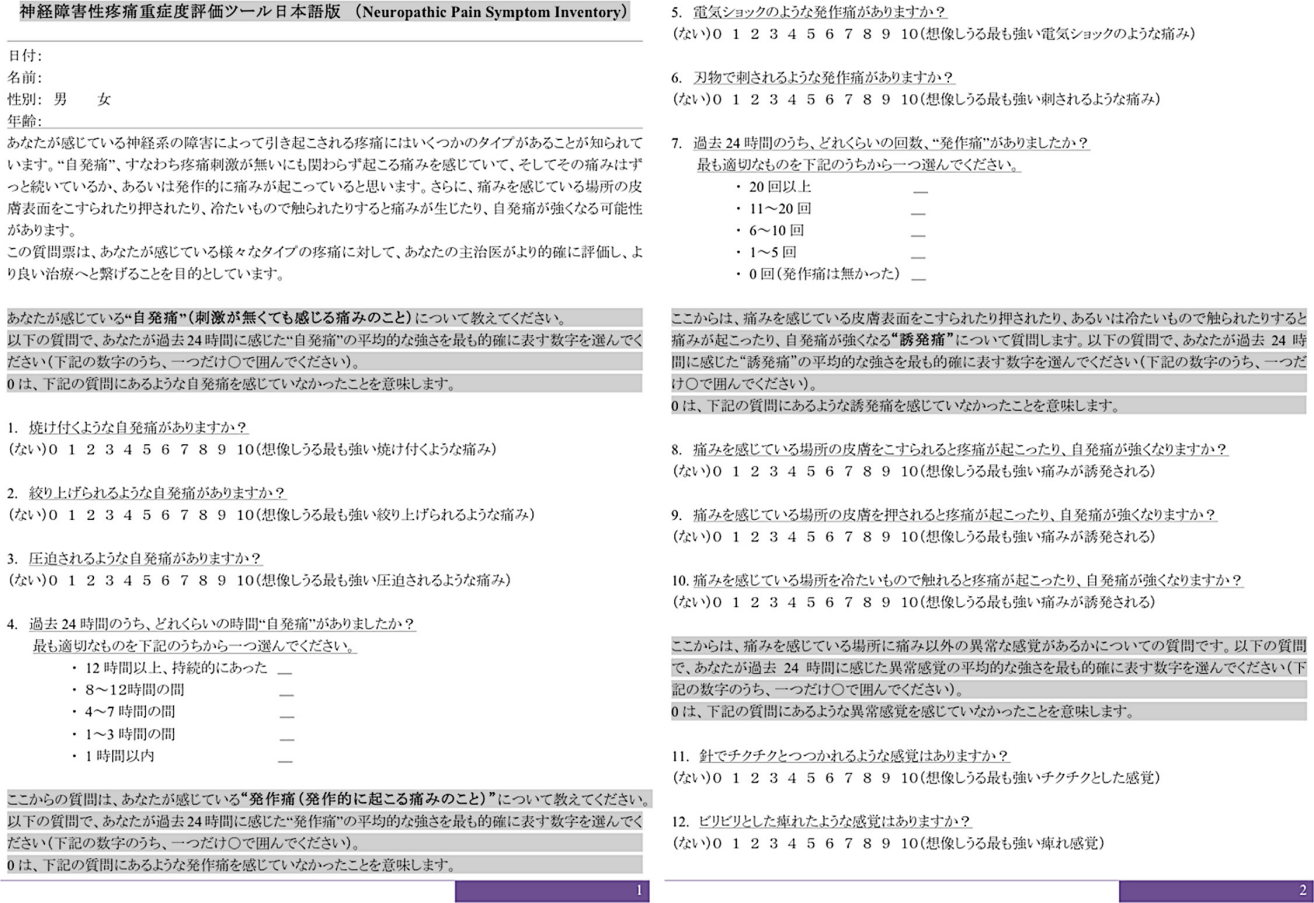
The Neuropathic Pain Symptom Inventory-Japanese Version (NPSI-J).

Various evaluation tools based on these characteristic descriptions have been developed to identify the particular mechanisms of neuropathic pain in clinical settings. Practically, several self-administered questionnaires have been established to evaluate the characteristics of neuropathic pain, including the Neuropathic Pain Scale [7] and Neuropathic Pain Symptom Inventory (NPSI) [8]. The NPSI is a self-administered questionnaire specifically designed to evaluate the different positive symptoms of neuropathic pain. This questionnaire has already been translated and validated into the German [9], Italian [10], Portuguese [11], and Spanish languages [12]. The original authors had also prepared a Japanese version of the NPSI, although some of the descriptions were unnatural according to native Japanese speakers. Accordingly, the Japanese version of the NPSI (NPSI-J) was translated and cross-culturally adapted into Japanese for academic use, under license by the original researchers according to established guidelines (Fig 1) [13, 14]. First, for forward translation, a professional native Japanese translator and bilingual Japanese physician independently translated the original NPSI into Japanese. Second, an expert committee, including specialists in pain management, orthopedics, and methodology, conducted a synthesis of this translation. Third, two native English translators who were uninformed about the nature of the study, completed back translations of the translated NPSI; these were subsequently sent to the expert committee to detect any existing cultural biases, after which the final version of NPSI-J was completed. As a result, the NPSI-J was approved through linguistic validation. Nevertheless, the psychometric validity of the NPSI-J has not yet been confirmed; therefore, this study aimed to assess the psychometric validity and reliability of the NPSI-J.

## Subjects and Methods

The study protocol was approved by the institutional review board of the Clinical Research Support Center, The University of Tokyo Hospital.

We conducted an observational study of enrolled patients who suffered from pain at an intensity of ≥3/10 on an 11-point numerical rating scale (NRS). We included patients with neuropathic pain diagnosed by a pain specialist in the pain center as per the guidelines established by the International Association for the Study of Pain (IASP) [3]. In the pain center, neuropathic pain patients with a stable disease condition and tolerable pain were selected; in addition, patients with little estimated change in pain during the study period were selected. Patients with cultural or language barriers, or poor mental health statuses that prevented them from understanding or responding to the proposed questions were excluded from this study. Written informed consent was provided by selected patients from both groups.

In the first survey, patients were asked to complete a set of questionnaires including the NPSI-J, a three-type numeric rating scale (NRS) of pain (i.e., pain during the survey, four-week average, and maximum pain experienced), and the Medical Outcomes Study 36-Item Short-Form Health Survey (SF-36). Patients answered questions regarding their demographic data (e.g., age, sex, height, weight, occupation, smoking history, and education). Thereafter, physicians reported the original diagnoses, comorbidities, and treatment options. The second survey questionnaire was administered to patients at 2–5 weeks after the first visit; this survey included the same set of three questionnaires with one additional question regarding whether an increase, decrease, or no change in pain had been observed since the administration of the first survey.

The NPSI comprises four main components that encompass 12 questions. In the first component, the patient is asked to identify the presence of spontaneous pain sensations on an 11-point (0–10) numerical scale (e.g., burning, squeezing, and pressure). In this component, the patient is also asked about the duration of spontaneous pain during the past 24 hours. In the second component, the patient is asked about paroxysmal pain, or sensations of an electric shock and stabbing, and is also asked about the times at which pain attacks occurred during the past 24 hours. In the third component, the patient is asked about evoked pain, or that provoked or increased by brushing, pressure, and contact with something cold. In the final component, the patient is asked about paresthesia/dysesthesia or sensations of pins and needles and/or tingling. The total score is calculated by adding the scores from the four components; as questions regarding the duration of spontaneous pain and the times at which pain occurred in the previous 24 hours are not included in the score, the total score represents the scores from 10 questions.

Pain intensity was assessed using a three-type NRS in which the patient was asked to grade the actual pain level experienced, the maximum pain level experienced in the last four weeks, and the average pain level experienced in the last four weeks on a scale of 0–10 (0 = no pain, 10 = worst pain imaginable).

The SF-36 comprises eight subscales: physical functioning, physical role functioning, bodily pain, general health perceptions, vitality, social role functioning, emotional role functioning, and mental health [15, 16]. Each subscale is transformed to a score ranging from 0 to 100, with lower scores indicating a poorer health-related quality of life. For this analysis, we used two summed scores: the Physical Component Score (PCS) and the Mental Component Score (MCS). Each score has the same mean and standard deviation (50 and 10, respectively) in a normal population.

### Validity

To establish construct validity, we performed an exploratory factor analysis with a principal components extraction. The Kaiser criterion (eigenvalues >1.0) and Scree plot were used to determine the number of factors. To determine criterion-related validity, we calculated the Pearson correlation coefficient between the NPSI-J, NRS, PCS (SF-36), and MCS (SF-36). The following ranges are generally accepted rankings for coefficients: 1.0–0.81 (excellent); 0.80–0.61 (very good); 0.60–0.41 (good); 0.40–0.21 (fair); and 0.20–0 (poor) [17].

### Reliability

Internal consistency was measured using Cronbach’s alpha. Alpha coefficients ≥0.70 were considered indicative of adequate scale reliability at the group comparison level [18]. Repeatability was assessed via a test–retest method. Neuropathic patients were subjected to retesting >2 weeks after the first survey. Intraclass correlation coefficients (ICCs) between the test and retest scores were calculated using data from patients who responded with no changes in symptoms between the two surveys; moreover, those with coefficients >0.80 were considered to have excellent reliability [19].

All statistical analyses were performed using the Statistical Package for the Social Sciences (SPSS version 21.0) software (SPSS, Inc., Chicago, IL, USA).

## Results

A total of 60 Japanese patients with neuropathic pain were recruited for this study. However, 4 patients were excluded because of incomplete responses to the proposed questions. Accordingly a total of 56 patients were evaluated further. The demographic characteristics of these patients are presented in Table 1. Specific etiologies of pain for patients were brachial plexus injury (11 patients), radiculopathy (11 patients), spinal cord injury (10 patients), herpes zoster (8 patients), diabetic or alcoholic polyneuropathies (7 patients), phantom pain (5 patients), complex regional pain syndrome (CRPS; 2 patients), carpal tunnel syndrome (1 patient), and thalamic pain (1 patient).

**Table 1:** Demographic data of study patients.

	Mean (SD)
**Age (mean)**	58.4 (15.2)
**Male/Female**	37/19
**Height (mean)**	163.9 (9.7)
**Weight (mean)**	64.0 (17.7)
**Duration (months)**	64.5 (73.3)

NeP: neuropathic pain, NocP: nociceptive pain, SD: standard deviation

Tables 2 and 3 presents the scores for each NPSI-J questionnaire, Pain intensity, and SF-36. Pain scores were relatively higher than past studies [9, 10].

**Table 2:** Neuropathic Pain Symptom Inventory Questionnaire-Japanese version (NPSI-J): summary of patient responses.

	Mean (SD)
Q1. Burning	5.1 (3.2)
Q2. Squeezing	3.8 (3.6)
Q3. Pressure	4.1 (3.4)
Q5. Electric shocks	3.5 (3.8)
Q6. Stabbing	2.9 (3.5)
Q8. Provoked by brushing	3.8 (3.7)
Q9. Provoked by pressure	3.7 (3.4)
Q10. Provoked by cold stimulation	3.5 (3.2)
Q11. Pins and needles	4.6 (3.5)
Q12. Tingling	6.9 (3.0)
Summary	43.3 (21.6)

SD, standard deviation

**Table 3:** Pain intensity, NPSI-J, and SF-36 scores.

	NeP (SD)
Pain Intensity-NRS (present)	6.6 (2.2)
Pain Intensity-NRS (average)	6.8 (1.8)
Pain Intensity-NRS (maximum)	8.3 (1.6)
NPSI	43.3 (21.6)
PCS (SF-36)	26.7 (16.5)
MCS (SF-36)	41.0 (11.9)

NeP: neuropathic pain, NocP: nociceptive pain, NRS: numerical rating scale, SF-36: the Medical Outcomes Study 36-Item Short-Form Health Survey, PCS: Physical Component Score, MCS: Mental Component Score

### Validity

The factor analysis with varimax rotation, calculated using the Kaiser criterion (eigenvalues ≥1.0) and Scree plot, revealed that the main component of the NPSI-J comprised three determinative factors. Factor 1 included four items: burning, electric shock, stabbing, and pins and needles, which can be named as superficial pain. Factor 2 included three items related to evoked pain: brushing, pressure, and contact with cold. Factor 3 included three items: squeezing, pressure, and tingling, which can be named as ongoing pain (Table 4). Regarding criterion-related validity, the total score of the NPSI-J exhibited statistically significant correlations with the pain intensity (Table 5). No significant correlation was observed between the NPSI-J and SF-36 scores.

**Table 4:** Rotated factor loadings of factor analysis.

	Factor 1	Factor 2	Factor 3
Q1. Burning	**0.511**	0.065	-0.089
Q2. Squeezing	-0.165	0.013	**1.081**
Q3. Pressure	0.267	-0.058	**0.467**
Q5. Electric shocks	**0.935**	-0.040	-0.047
Q6. Stabbing	**0.687**	-0.063	0.162
Q8. Evoked by brushing	0.017	**0.956**	0.028
Q9. Evoked by pressure	0.088	**0.916**	-0.028
Q10. Evoked by cold stimulation	-0.096	**0.668**	0.003
Q11. Pins and needles	**0.490**	0.063	0.304
Q12. Tingling	0.178	0.023	**0.386**

**Table 5:** Pearson correlation coefficient with NPSI-J.

	NPSI-J	P value
Pain-Intensity (NRS)	0.414	0.002
PCS (SF-36)	−0.058	0.67
MCS (SF-36)	−0.141	0.30

NRS: numerical rating scale, SF-36: the Medical Outcomes Study 36-Item Short-Form Health Survey, PCS: Physical Component Score, MCS: Mental Component Score

### Reliability

The Cronbach alpha value for the main component of NPSI-J (i.e., “burning sensation”) was 0.863, which was comparable to the values of 0.75 and 0.80 reported for the German and Spanish versions, respectively [9, 12]. All of the values of Cronbach alpha if delete each items were ≤0.857. The score for each of the 10 questions in the NPSI-J exhibited a statistically significant correlation with the total NPSI-J score.

We were able to recruit 16 patients with neuropathic pain for a test–retest study; of these, data from 11 patients who reported no changes in their symptoms were evaluated. The average interval between the two surveys was 23.1 days [standard deviation (SD): 8.3]. The mean scores of the first and second surveys were 44.9 (SD: 20.0) and 43.9 (SD: 22.8), respectively. Furthermore, the ICC between the two scores was 0.81, an excellent value despite the relatively long interval between the two surveys (Table 6).

**Table 6:** Intraclass correlation coefficients (ICC) of questionnaires between the first and second visits.

	ICC
Q1. Burning	0.67
Q2. Squeezing	0.79
Q3. Pressure	0.93
Q5. Electric shocks	0.99
Q6. Stabbing	0.92
Q8. Evoked by brushing	0.88
Q9. Evoked by pressure	0.66
Q10. Evoked by cold stimulation	0.79
Q11. Pins and needles	0.84
Q12. Tingling	0.93
Total score	0.81

## Discussion

The results of the present study revealed moderate-to-good psychometric validity and reliability for the NPSI-J, and were comparable with results obtained in previous studies [8–11]. Regarding the validity of this tool, a factor analysis revealed that the 10 Likert items of the NPSI-J comprised three determinative factors that could be designated as “superficial pain,” “evoked pain,” and “ongoing pain.” These factors are consistent with the clinical characteristics of neuropathic pain. Furthermore, regarding the criterion-related validity, the correlations between the NPSI-J and NRS were moderate, indicating that the NPSI-J can reflect pain intensity. Additionally, the NPSI-J demonstrated fair to good criterion-related validity, excellent internal consistency, and statistically significant good reliability in this study, although the number of the patients was limited, particularly with regard to the repeatability analysis. Because this study evaluated various types of neuropathic pain, the methods and results obtained herein might be useful in a wide range of patient populations suffering from neuropathic pain.

Early work by Dubuisson and Melzack [20] and later work by Boureau et al. [21] proposed anecdotal opinions that characteristic pain descriptions might discriminate against neuropathic pain; also, as these descriptions might result from particular mechanisms, specific management strategies could be applied. The use of some types of treatment modalities (e.g., pharmacotherapy, neurorehabilitation, and neuromodulation) clearly revealed that different pain-analgesic effects are observed for different pain characteristics; in other words, the analgesic effect depends on the pain characteristics [22–25]. For example, the ongoing, paroxysmal, and evoked pain characteristics measured on the NPSI would be sensitive to repetitive transcranial magnetic stimulation (rTMS), whereas dysesthesia/paresthesia is seldom sensitive to rTMS in patients with neuropathic pain [25]. In other instances, in patients with phantom limb pain, mirror visual feedback treatment has been shown to improve the characteristic features of pain, which have been spontaneously described to associate with deep tissue-mediated pain (e.g., pressing, twisting, and cramp-like), but was not found to improve the characteristics of pain associated with the application of noxious stimuli to the skin surface (e.g., burning, stabbing, and pins and needles) [24]. Furthermore, minocycline was found to improve the affective characteristics evaluated on the McGill Pain Questionnaire [26], but did not alleviate the sensory characteristics [22]. In particular, a recent report involving the NPSI successfully demonstrated that the identification of subgroups of patients with distinct neuropathic pain characteristics should be encouraged in order to predict their differential responses to various pharmacotherapies, thereby indicating that heterogeneity among patient populations should be considered to allow a more stratified or even personalized treatment approach according to the pain characteristics [23].

Different sets of pain description questionnaires in various languages have been used worldwide. A single common set of pain description questionnaires specifically designed for neuropathic pain assessment on a global scale, would be a truly useful tool that would allow comparisons of reports regarding the relationships between treatments and pain characteristics, and would improve clinical outcomes in general practice. Because the NPSI is, to our best knowledge, the most globally available instrument for measuring neuropathic pain characteristics (i.e., available in English, French, German, and Portuguese), we selected this tool to share and compare the varied outcomes of clinical studies conducted in Japan and other linguistic areas. For example, regarding pharmacotherapy for neuropathic pain, some guidelines have proposed the use of first-, second-, and sometimes third-line analgesics. Several analgesics are usually included in these respective lines, but the selection criteria for analgesics in each line are rarely proposed. The identification of subgroups of patients with distinct neuropathic pain characteristics and differential responses to varied pharmacotherapies could be performed worldwide using a common tool, such as the NPSI, and would thus provide a global cross-reference.

## Conclusion

This study confirms that the NPSI-J, which enables physicians to precisely investigate neuropathic pain, is a reliable and valid pain assessment tool. This tool allows us to understand differences in the neuropathic pain components associated with each type of neuropathic disease and to determine the responses of each neuropathic pain component to various treatments, allowing us to select the best treatment options for a range of painful neuropathic diseases.
